# Extracranial arteriovenous malformations demonstrate dysregulated TGF-β/BMP signaling and increased circulating TGF-β1

**DOI:** 10.1038/s41598-022-21217-0

**Published:** 2022-10-05

**Authors:** Ting Wei, Gresham T. Richter, Haihong Zhang, Ravi W. Sun, Conor H. Smith, Graham M. Strub

**Affiliations:** 1 Arkansas Children’s Research Institute, 13 Children’s Way, Little Rock, AR 72202 USA; 2 Department of Otolaryngology, University of Arkansas for Medical Sciences, 4301 W. Markham St., Little Rock, AR 72205 USA

**Keywords:** Molecular medicine, Growth factor signalling

## Abstract

Extracranial arteriovenous malformations (AVMs) are characterized by anomalous arterial-to-venous connections, aberrant angiogenesis, local inflammation and hypoxia, and disorganized histological architecture; however, the precise molecular perturbations leading to this phenotype remain elusive. We hypothesized that extracranial AVM tissue would demonstrate deregulation of the TGF-β/BMP signaling pathway, which may serve as a potential target in the development of molecular-based therapies for AVMs. AVM tissue was harvested during resection from 10 patients with AVMs and compared to control tissue. Blood was collected from 14 AVM patients and 10 patients without AVMs as controls. Expression of TGF-β/BMP pathway components was analyzed using RT-PCR, western blotting, and immunohistochemistry. Circulating levels of TGF-β1 were analyzed by ELISA. Paired *t* tests were utilized to perform statistical analysis. The mRNA levels of TGF-β1, ALK1, Endoglin (ENG), Smad6, Smad7, and Smad8 were significantly elevated in AVM tissue when compared to controls. Protein levels of TGF-β1 and Smad3 were elevated in AVM tissue while protein levels of BMP-9, ALK1, Smad1, Smad6, and Smad8 were significantly decreased in AVMs. Immunohistochemistry demonstrated increased TGF-β1 in the perivascular cells of AVMs compared to normal controls, and circulating levels of TGF-β1 were significantly higher in AVM patients. Patients with AVMs demonstrate aberrant TGF-β/BMP expression in AVM tissue and blood compared to controls. Targeting aberrantly expressed components of the TGF-β/BMP pathway in extracranial AVMs may be a viable approach in the development of novel molecular therapies, and monitoring circulating TGF-β1 levels may be a useful indicator of treatment success.

## Introduction

Arteriovenous malformations (AVMs) are high-flow vascular lesions composed of a network of direct arterial-to-venous connections suspected to be devoid of a normal capillary bed with impaired exchange of nutrients and oxygen. AVMs are congenital lesions affecting 1:100,000 patients^1^. Extracranial (peripheral) AVMs can be complex and infiltrative lesions that are often congenital and characterized by unchecked vascular expansion and soft tissue destruction. Histologically, AVMs demonstrate abnormal perivascular tissue, disorganized vascular channels and evidence of disrupted vascular remodeling. This leads to thin, leaky and friable vessels that tend to bleed, either spontaneously or during interventions^2^. Despite currently available surgical and endovascular techniques to treat these lesions, their recurrence rate exceeds that of most cancers^3^ and leads to significant functional and aesthetic morbidities^4^. Because of the infiltrative nature of these lesions, they are almost never “cured;” the goal of treatment is typically control of growth and bleeding^5^. Although the exact mortality and cost associated with AVM treatment is not known, patients typically require multiple treatment interventions throughout their lifetime. These challenges necessitate the development of topical or systemic treatment modalities that target the aberrant pro-angiogenic signaling cascades that have been demonstrated in these lesions.

The etiology of extracranial AVMs is poorly understood, although some hereditary conditions with AVM have been described. These include hereditary hemorrhagic telangiectasia (HHT) and capillary malformation-arteriovenous malformation (CM-AVM) which have been attributed to inherited mutations in RASA1, endoglin, and activin receptor-like kinase 1 (ALK1). However, most extracranial AVMs are sporadic and likely the result of somatic mutations (reviewed by Cunha et al.^6^). For example, somatic mutations of MAP2K1 have been reported in sporadic extracranial AVMs^7^, as have mosaic-activating variants in the components of RAS/MAPK signaling (KRAS, BRAF, and MAP2K1)^8^. While these mutations offer insights into the proliferative and pro-angiogenic nature of AVMs, they do not fully explain the dysmorphic vascular architecture of these lesions. Histologically, the AVM phenotype suggests a disordered process of vascular remodeling and differentiation, processes directly regulated by the transforming growth factor (TGF)-β/bone morphogenetic protein (BMP) superfamily^9^.

The highly conserved TGF-β/BMP signaling pathway is complex and involves multiple ligands, second messengers, and feedback mechanisms^10,11^. In endothelial cells (ECs), the secreted ligands TGF-β1 and BMP-9 initiate signaling via recruitment of two specific serine/threonine kinase heterotrimeric complexes, ALK1 and ALK5^12–14^. These complexes then recruit and activate Smad signaling effectors (Smads1, 5, and 8 from ALK1 activation and Smads 2 and 3 from ALK 5 activation), which complex with Smad4 and translocate to the nucleus, where various endothelial-specific genes are then activated^15^. Concurrently, inhibitory Smads 6 and 7 are activated by ALK1 and ALK5 activation, serving as a negative feedback control of this pathway^16^. The balance of this intricate signaling cascade is critically important for normal vascular development, maintenance of vascular permeability, and vascular remodeling, all of which are abnormal in extracranial AVMs.

Disruption of the TGF-β/BMP pathway has been observed in other vascular malformations, including HHT and cerebral cavernous malformations (CCM), which can present with AVMs or vascular cavernomas^6^. ALK1, endoglin, and BMP-9 mutations have been described in the affected tissues of patients with HHT^17–19^ but not in sporadic extracranial AVMs. Endothelial cells carrying any of the known CCM mutations also demonstrate dysregulated TGF-β/BMP signaling^20,21^. Given these observations, several strategies targeting this pathway in HHT and CCM have been employed with varying degrees of success, including anti-fibrinolytics, anti-estrogens, chemotherapeutic agents, and TGF-β receptor antagonists^20,22–25^.

The potential of targeting the TGF-β/BMP pathway in sporadic extracranial AVMs remains unknown. The purpose of this study was to determine if extracranial AVMs exhibit aberrant TGF-β/BMP signaling and thus possibly identify a molecular pathway that could be targeted with new therapies.

## Methods

### Specimen collection and preparation

This study was approved by the Institutional Review Board of the University of Arkansas for Medical Sciences (#228710). Written informed consent was obtained from the participants or their guardians prior to tissue and serum collection. All described experiments were performed in accordance to the relevant guidelines and regulations set forth by the IRB. Human AVM tissues (n = 10, ages 5 months–2.5 years), normal tissues (n = 10, ages 2 months–19 years), AVM patient sera (n = 14, ages 20 months–19 years), and normal control sera (n = 10, ages 14 months–34 years) were collected from both pediatric and adult patients after induction of general anesthesia for excisions. AVM tissue was collected during AVM resection, and normal soft tissue (skin and subcutaneous tissue) was collected from otherwise healthy patients undergoing excisions of other vascular lesions such as infantile hemangioma from regions distant from their respective lesions. Patient serum was prepared from whole blood collected in BD Vacutainer SSD tubes with silica clot activator and separating polymer gel. Histological examination by a pathologist experienced with vascular anomalies confirmed the diagnosis of AVM by H&E. After surgical harvest, tissue specimens were divided and immediately put into either 10% neutral formalin for histological staining or directly frozen on dry ice and transferred to a − 80 °C freezer for western blotting or RT-PCR. Serum specimens were centrifuged for 15 min at 1000*g* at room temperature (RT), and supernatants were aspirated and transferred to a − 80 °C freezer for storage until analysis.

### Real-time PCR

Thirty milligrams of sample tissue was used for RNA isolation with the RNeasy Plus Kit (Qiagen) according to the manufacturer’s instructions. RNA samples were reverse-transcribed into cDNA with the TaqMan Reverse Transcription Reagents Kit (Thermofisher) using random primers according to the manufacturer’s instructions. The real-time PCR amplifications were performed on an ABI 7900HT System. Thermal cycling conditions were: 1 cycle of 50 °C for 2 min, then 1 cycle of 95 °C for 10 min, followed by 40 cycles of 95 °C for 15 s and 60 °C for 1 min. The total reaction volume of 10 µl contained 5 ng of cDNA template, 5 µl of 2 × PCR Master Mix (Thermofisher), and 0.5 µl of 20 × primers. RT-PCR primers for TGF-β1 (Hs00998133_m1), ALK5 (Hs00610320_m1), ALK1 (Hs00953798_m1), BMP-9 (Hs00211913_m1), SMAD1 (Hs00195432_m1), SMAD2 (Hs00183425_m1), SMAD3 (Hs00969210_m1), SMAD4 (Hs00929647_m1), SMAD5 (Hs00195437_m1), SMAD6 (Hs00178579_m1), SMAD7 (Hs00998193_m1), and SMAD8 (Hs00195441_m1) were purchased from Thermofisher Scientific. The eukaryotic 18S rRNA (Thermofisher, Hs99999901_s1) was used as the endogenous control. The comparative Ct method was used to determine relative quantification.

### Western blot analysis

Total protein was extracted using T-PER tissue protein extraction reagent (Thermo Scientific) supplemented with Halt Protease Inhibitor Cocktail (Thermo Scientific) and 1 mM EDTA (Invitrogen). Protein concentrations were measured using a BCA protein assay kit (Thermo Scientific). Total protein (10–20 µg) was loaded onto NuPAGE^®^ Novex^®^ 4–12% Bis–Tris Protein gels (Invitrogen) for electrophoresis under reducing condition and transferred to PVDF membranes (Bio-Rad). The membranes were blocked with 5% non-fat milk in TBST (Thermo Scientific) for 1 h at RT, followed by incubation with primary antibodies at 4 °C overnight. For certain experiments, membranes were cut at specific molecular weight levels to enable the same gel to be incubated with multiple primary antibodies. Blots were then washed with TBST three times, followed by incubation with horseradish peroxidase–conjugated secondary antibodies (Santa Cruz, 1:2000) for 1 h at RT. Blots were developed with the Novex ECL Chemiluminescent Substrate Reagent Kit (Invitrogen) for 1 min in dark and exposed on Image Quant™ LAS 4000 (GE Healthcare). The primary antibodies used were TGF-β1 (Abcam ab27969, 1:2000), ALK5 (Abcam ab51871, 1:200), BMP-9 (Santa Cruz sc-514211, 1:200), ALK1 (Abcam ab68703, 1:1000), Smad1 (Cell signaling #9743, 1:1000), Smad2 (Cell signaling #5339, 1:1000), Smad3 (Cell signaling #9523, 1:1000), Smad4 (Abcam ab130242, 1:2000), Smad5 (Cell signaling #9517, 1:1000), Smad6 (Abcam ab110156, 1:1000), Smad7 (Novus 293039, 1:100), Smad8 (Santa Cruz sc-7442, 1:200), GAPDH (Cell signaling #2118, 1:10,000), and α-Tubulin (Cell signaling #2125, 1:1000). Images were captured and quantitated using Image Quant TL 7.0 software (GE Healthcare). All full length gels as well as gels that were cut prior to primary antibody incubation are included in Supplementary Material.

### Immunohistochemistry

After deparaffinization and rehydration, sections were heated to 95 °C for 15 min in Citrate Buffer, pH 6.0 (Invitrogen), for antigen retrieval. Hydrogen peroxide 3% (Fisher Scientific) was used to block endogenous peroxidase activity for 15 min at RT. Sections were preincubated with normal serum for 20 min at RT, then incubated in primary antibody TGF-β1 (Abcam ab66043, 1:200) at 4 °C overnight. After washing in PBST, sections were incubated in biotinylated secondary antibody (Vector Labs, 1:200) for 30 min at RT followed by ABC reagent (Vector labs) for 30 min. Color was developed using ImmPACT™ DAB (Vector Labs). The sections were then counterstained with hematoxylin (Fisher Scientific), dehydrated, and mounted using permanent mounting media (Fisher Scientific). Slides with no primary antibody applied were used as negative controls. The staining results were validated by a blind review performed by a pathologist with extensive experience examining vascular anomalies and immunohistochemistry. Microscopy was performed and images were taken with a Biotek Cytation5 Imaging Reader (Agilent Technologies, California, USA) and Olympus BX43 light microscope with Infinity 3S camera (Olympus, Pennsylvania, USA), respectively.

### Enzyme-linked immunosorbent assay

Serum concentration of TGF-β1 was determined using a Human TGF-β1 Quantikine ELISA Kit (R&D Systems, DB100B) according to the manufacturer’s instructions. Optical density was read on an iMarkTM microplate absorbance reader (Bio-Rad).

### Statistical analysis

Normalized RT-PCR values, quantitation results of western blot bands, and concentrations of soluble protein on ELISA for the AVM and control groups were compared, and a paired *t* test was used to determine statistical significance. The statistical analysis was run on an IBM SPSS Statistics 22. *P* < 0.05 was considered statistically significant. Asterisks (*P < 0.05, **P < 0.01, ***P < 0.001, ****P < 0.0001) are used to indicate levels of significance in figures.

## Results

### mRNA expression of TGF-β/BMP pathway components is dysregulated in AVM tissue

**Figure 1:**
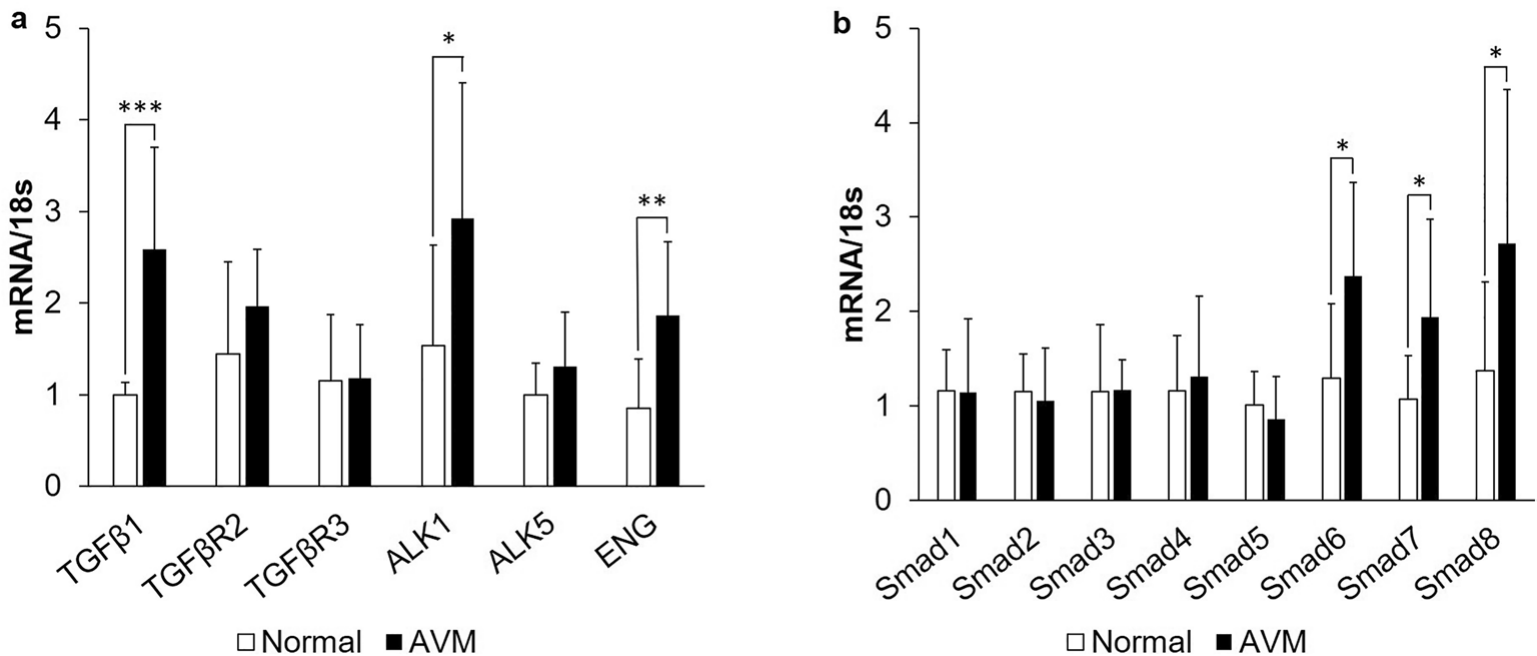
mRNA levels of TGF-β/BMP pathway components are dysregulated in AVM tissue. (**a**) mRNA expression of TGF-β1 ligand, the TGF-β receptors TGFβR2 and TGF-βR3, ALK1 and ALK5, and the accessory receptor endoglin (ENG) were analyzed in AVM tissue (n = 10 patients, black bars) and normal tissue (n = 10 patients, white bars). (**b**) mRNA expression of Smads 1–8 were analyzed in AVM tissue (n = 10 patients, black bars) and normal tissue (n = 10 patients, white bars). Relative levels of mRNAs were normalized to the expression of total 18s RNA. Error bars represent standard deviation. Paired *t* tests were performed to determine statistical significance (asterisks: *P < 0.05, **P < 0.01, ***P < 0.001).

mRNA expression of TGF-β/BMP pathway components in AVM tissues (n = 10) and normal tissues (n = 10) is shown in Fig. 1. mRNA levels of TGF-β1 ligand, the serine/threonine kinase receptor ALK1, and the accessory receptor ENG were significantly elevated in AVM tissue compared with controls (*P* = 0.001, *P* = 0.016, and *P* = 0.001, respectively) (Fig. 1a). mRNA levels of the signaling effectors Smad6, Smad7, and Smad8 were also upregulated in AVM tissue when compared with controls (*P* = 0.033, *P* = 0.038, and *P* = 0.037, respectively) (Fig. 1b). mRNA levels of the TGF-β receptors TGF-βR2 and TGF-βR3, ALK5, and signaling effectors Smads1–5 were not statistically different between AVM and control tissues. BMP-9 mRNA was undetectable in both AVM and control tissues (data not shown). All target gene mRNA levels were normalized to mRNA levels of the eukaryotic 18S rRNA.

### Levels of TGF-β/BMP pathway component proteins are dysregulated in AVM tissue

**Figure 2:**
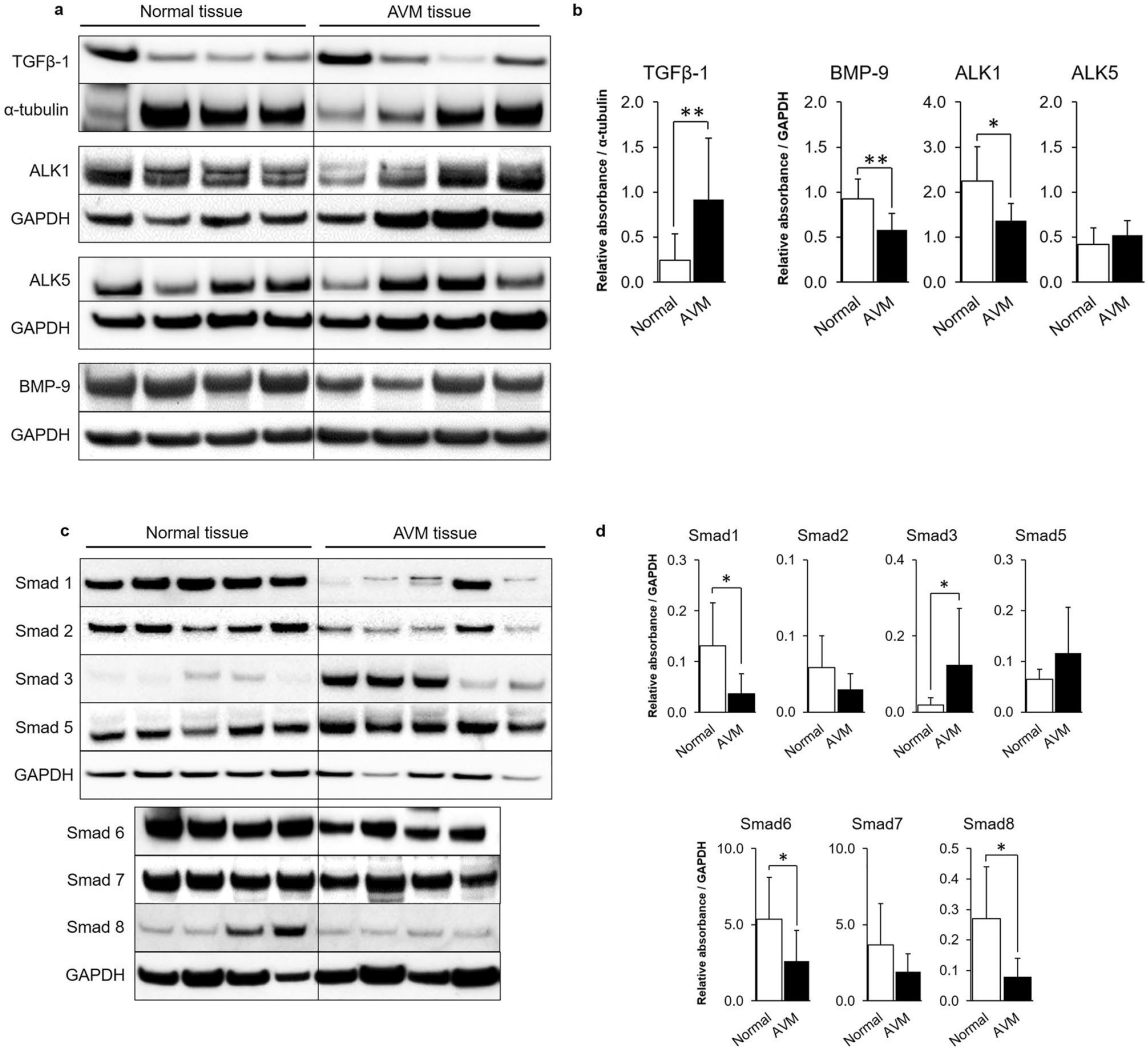
Expression of TGF-β/BMP pathway component proteins are dysregulated in AVM tissue. Protein levels of TGF-β1, ALK1, ALK5, and BMP-9 (**a**) and of Smad1–8 (**c**) were analyzed by western blot from AVM tissue samples (n = 10 patients) and normal tissue samples (n = 10 patients). Representative examples from each group are illustrated. GAPDH and α-tubulin protein expression were determined as loading controls. The total density of each protein band (n = 10 AVM and n = 10 normal) was determined using an ImageQuant developer and software, and relative expression was calculated by normalizing to loading controls (**b,d**). Error bars represent standard deviation. Paired *t* tests were performed to determine statistical significance (asterisks: *P < 0.05, **P < 0.01, ***P < 0.001).

Protein levels of TGF-β/BMP pathway components in AVM tissues (n = 10) and normal tissues (n = 10) and representative western blots are shown in Fig. 2. Protein levels of TGF-β1 ligand was significantly elevated in AVM tissues when compared to controls (*P* = 0.006) while levels of BMP-9 ligand protein were significantly reduced (*P* = 0.004) (Fig. 2a,b). Receptor ALK1 protein was significantly decreased in AVM tissue (*P* = 0.015) while ALK5 protein levels were similar between AVM and control tissue (Fig. 2a,b). Protein levels of TGF-β/BMP pathway signaling effectors Smad1, Smad6, and Smad8 were significantly decreased in AVM tissues (*P* = 0.050, *P* = 0.015, and *P* = 0.014, respectively) while Smad3 protein levels were significantly elevated in AVM tissues (*P* = 0.031) (Fig. 2c,d). Protein levels of Smad2, Smad5, and Smad7 were not significantly different between AVM and control tissues.

### TGF-β1 protein is elevated in AVM perivascular smooth muscle cells and in the circulation of AVM patients

**Figure 3:**
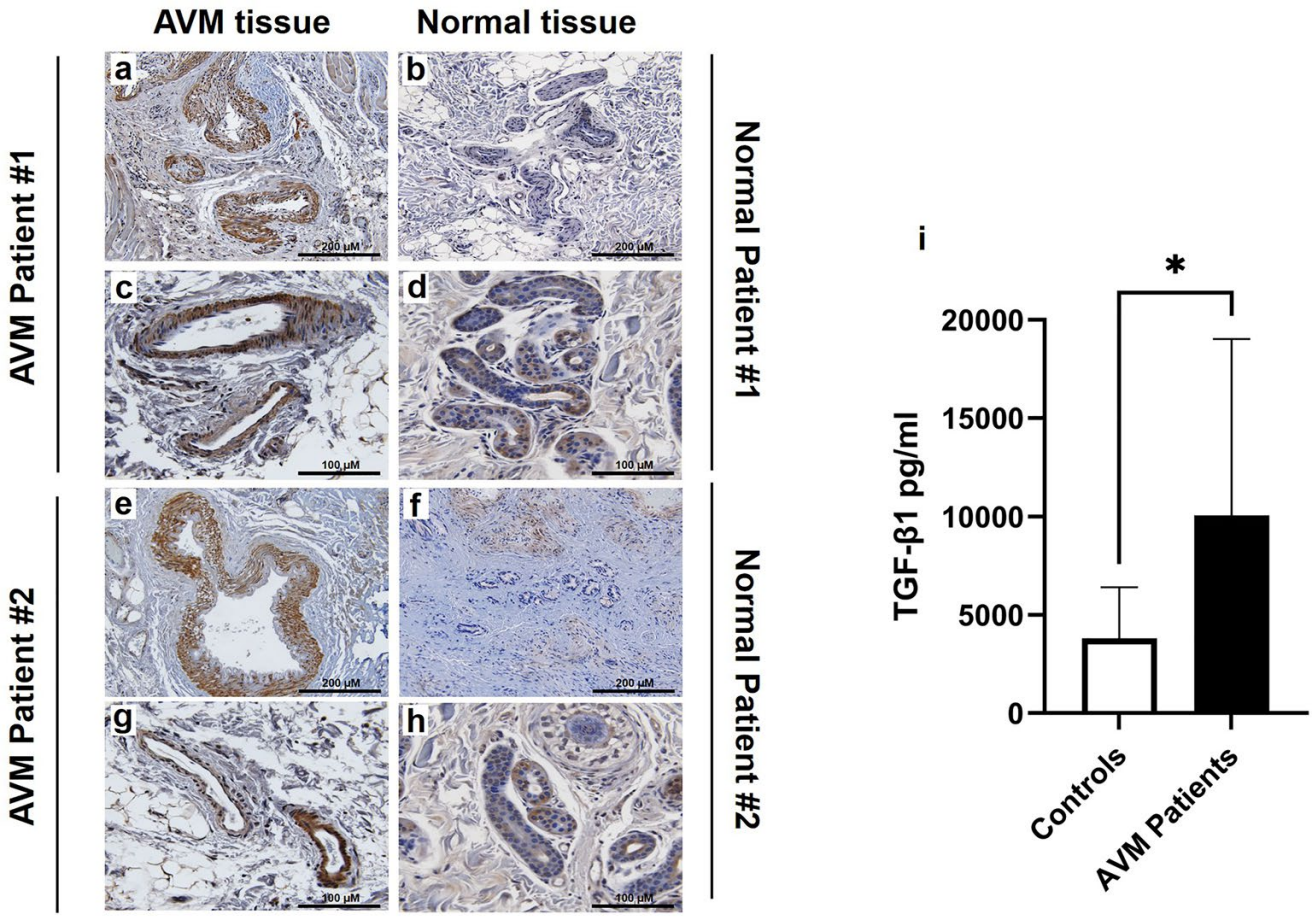
TGF-β1 protein is elevated in AVM perivascular smooth muscle cells and in the circulation of AVM patients. Paraffin-embedded slides prepared from tissue from 2 AVM patients (**a**,**c**,**e**,**g**) and 2 non-AVM patients (**b**,**d**,**f**,**h**) were stained for TGF-β1 protein and photographed under low- and high-power magnification. (**i**) Serum was isolated from blood samples collected from 14 AVM patients and 8 patients without AVMs, and ELISA was performed to determine the total levels of circulating TGF-β1. Error bars represent standard deviation. Paired *t* tests were performed to determine statistical significance (asterisks: *P < 0.05, **P < 0.01, ***P < 0.001).

The level of TGF-β1 protein in tissues and in serum of AVM patients and normal controls is displayed in Fig. 3. Representative sections of paraffin-embedded AVM tissues (n = 2) and normal control tissues (n = 2) are illustrated in Fig. 3a–h. AVM tissues demonstrated extensive TGF-β1 staining in perivascular cells while TGF-β1 was barely detectable or absent in all normal control tissues. Circulating levels of TGF-β1 in serum collected from AVM patients (n = 14) and patients without AVMs (n = 8) are displayed in Fig. 3i. The average level of circulating TGF-β1 was significantly higher in AVM patients (10,075.3 pg/ml) when compared to normal controls (3809.7 pg/ml) (*P* = 0.032).

## Discussion

This study presents evidence that an imbalance of TGF-β/BMP signaling, a critical pathway for maintenance of vascular integrity, is present in extracranial AVM tissue. In addition, we provide evidence of increased levels of TGF-β1 ligand in both the perivascular cells of AVMs and in the circulation of AVM patients, indicating that TGF-β1 may be a useful biomarker for therapeutic monitoring.

TGF-β/BMP signaling is a complex process involving a multitude of ligands, receptors, second messengers, and transcriptional targets. Adding to the complexity of this pathway is its apparent context-dependency: in certain cell types and conditions TGF-β/BMP signaling is pro-angiogenic whereas in other contexts the pathway inhibits angiogenesis^13,26^. Imbalance within the various cascades stimulated by TGF-β/BMP signaling leads to alterations in vascular integrity, endothelial cell function and migration, and angiogenic drive, all of which are abnormal in extracranial AVMs. The current study demonstrates that in AVM tissue, protein and mRNA levels of key TGF-β/BMP signaling components are imbalanced when compared to normal tissue—specifically, the signaling of BMP-9 and TGF-β1 through the receptors ALK1 and ALK5, respectively.

Binding of BMP-9 to ALK1 stimulates the phosphorylation of the receptor Smads 1, 5 and 8, which then complex with Smad4 and translocate to the nucleus^15^. This complex regulates several genes crucial for vascular integrity, including Notch pathway genes *Hes1, Hey1, Hey2*, and *Jag1*^27,28^ as well as the inhibitory Smad6, which regulates ALK1 expression^29^. Loss of ALK1 in mice leads to venous enlargement, vascular hyperbranching, and AVM formation via decreased Smad1/5/8 activity^30^. In our study, the protein levels of BMP-9, ALK1, Smad1, Smad6, and Smad8 were all significantly decreased in AVM tissue. Interestingly, mRNA levels of ALK1, Smad6, and Smad8 were increased in AVM tissue while mRNA levels of Smad1 remained unchanged. These data suggest that in AVM tissue, signaling through BMP-9/ALK1 is impaired, and a compensatory feedback loop increasing mRNA components of this pathway is disrupted.

**Figure 4:**
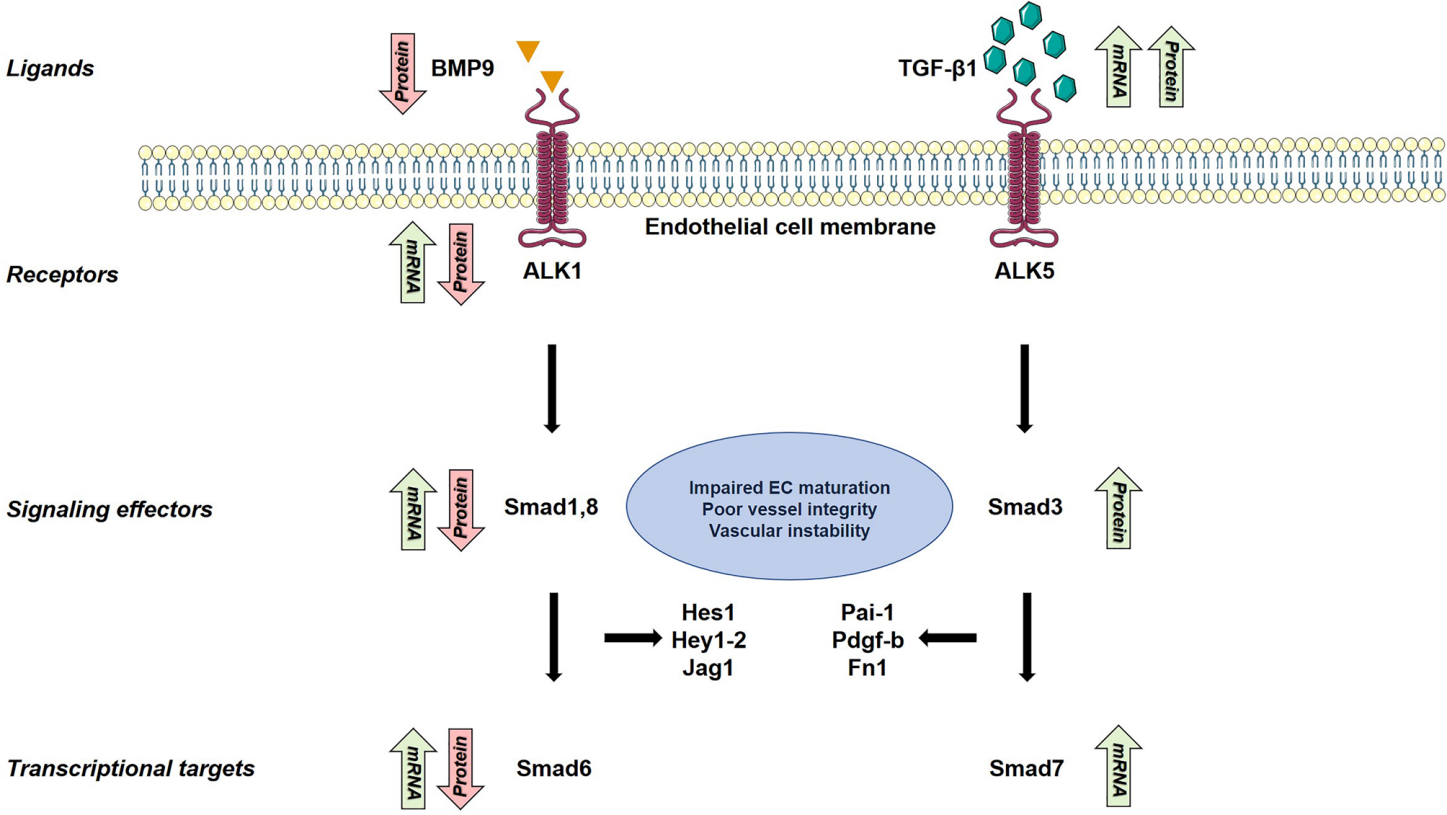
Schematic of dysregulated TGF-β/BMP expression in AVM endothelial cells. Levels of both circulating and intracellular TGF-β1 were elevated in AVM patients when compared to controls while intracellular BMP-9 levels were lower in AVM patients. Protein levels of components of the ALK1 → Smad1/5/8 → Smad6 pathway were decreased in AVM patients despite an increase in mRNA levels while Smads downstream of TGF-β1 → ALK5 activation demonstrated normal mRNA expression and elevated protein levels.

Our study also demonstrates a significantly elevated level of both TGF-β1 protein and mRNA in AVM tissue. TGF-β1 binds to ALK5, which was not differentially expressed at the mRNA or protein level. The downstream effector of ALK5, Smad3, was also overexpressed at the protein level in AVM tissue compared to normal controls. Genes regulated by Smad2/3 activation include plasminogen activator inhibitor 1 (*Pai-1*), platelet derived growth factor b (*Pdgf-b*), fibronectin (*Fn1*), and the inhibitory Smad7^31^. These genes play several critical roles in cell migration, clotting, senescence, wound healing, and maintenance of the extracellular matrix^32–39^. Taken together, our data suggest an imbalance between BMP-9/ALK1 and TGF-β1/ALK5 signaling may contribute to the multitude of impaired cellular functions present in extracranial AVMs (Fig. 4).

The etiology of extracranial AVMs remains under investigation but has been attributed to somatic mutations in several genes, including *MAP2K1* (*MEK1*), *KRAS*, and *BRAF*^40^. Several studies have demonstrated cross-talk between the expression of these genes and TGF-β/BMP signaling, which may suggest an underlying mechanism of altered TGF-β/BMP signaling in extracranial AVMs. Constitutively activated *MEK1* stabilizes the TβRII receptor, leading to increased signaling and decreased sensitivity to TGF-β-mediated growth suppression^41,42^. Oncogenic RAS can directly phosphorylate Smads 2 and 3, inhibiting their nuclear accumulation and ability to modulate downstream gene transcription^43^. Oncogenic BRAF mutations also activate TGF-β signaling, which, in turn, activates genes that drive endothelial-to-mesenchymal transition (EMT)^44^. The possible relationship between these causative somatic mutations in AVMs and imbalanced TGF-β/BMP signaling has not yet been elucidated.

Although the effect of local increased TGF-β1 production and release into circulation is unknown in AVMs, it may contribute to local inflammation, fibrosis, and smooth muscle proliferation. Overexpression of TGF-β1 in the lungs of fetal monkeys leads to pulmonary fibrosis and hyperproliferation of myofibroblasts^45^. During kidney ischemia, epithelial cells increase TGF-β1 secretion, which stimulates pericyte-to-mesenchymal cell transition, proliferation, and fibrosis that was reversible with anti-TGF-β1 antibody or TGF-βR inhibitor^46^. Increased circulating levels of TGF-β1 are associated with chronic kidney disease progression and scleroderma^47,48^ while TGF-β1 levels in HHT patient circulation are decreased^49^. In cancer, increased levels of circulating TGF-β1 are associated with increased metastasis, advanced TNM stage, and poorer prognosis^50–52^. As systemic molecular therapies are developed in the treatment of extracranial AVMs, monitoring levels of circulating TGF-β1 may be a useful indicator of treatment success, although whether this increase in circulating TGF-β1 is due to release from AVM tissue or from elsewhere in AVM patients has yet to be specifically demonstrated.

The promiscuousness of TGF-β/BMP deregulation in cancer, inflammation, fibrosis, and aberrant angiogenesis has led to the development of numerous therapeutic agents targeting this pathway (reviewed by Akhurst^53^ and Cunha^6^), including small molecule inhibitors, monoclonal antibodies, ligand traps, and anti-sense RNAs. Similar approaches have been used to target related signaling cascades in vascular anomalies. For example, sirolimus (rapamycin) has been demonstrated to be an effective agent in the treatment of lymphatic malformations by targeting the PIK3CA pathway, which is activated in the endothelial cells of these lesions^54,55^. ALK1 deletion activates the PI3K pathway in HHT mouse models that form AVMs, a process reversible by pharmacologic inhibition of PI3K^56^. FK506 (tacrolimus) increases ALK1 expression in ECs and restores TGF-β/BMP signaling in pulmonary hypertension^23,57^. MicroRNA-27b positively regulates TGF-β–induced endothelial-to-mesenchymal transition in ECs, which is reversible with antisense miR-27^58^. The effectiveness of these approaches in restoring normal TGF-β/BMP signaling in ECs isolated from extracranial AVMs should be evaluated in future studies.

## Conclusion

Extracranial AVM tissue demonstrates deregulation of TGF-β/BMP pathway components, characterized by decreased protein levels of BMP-9, ALK1, and Smads 1, 6, and 8 and increased TGF-β1 and Smad3 proteins. ALK1 and Smad6–8 mRNA levels are increased in AVMs, suggesting a post-transcriptional mechanism underlying their altered expression. Patients with extracranial AVMs also have significantly higher circulating levels of TGF-β1. Targeting components of the TGF-β/BMP pathway in extracranial AVMs may offer a novel therapeutic option, and monitoring circulating TGF-β1 may be a useful indicator of treatment response.

## Supplementary Information

Supplementary Figures.

## Data Availability

All available data is included in this manuscript. Full images of gels/blots are included in supplementary material. No data representing proteomics, protein sequences, DNA/RNA sequences, genetic polymorphisms, linked genotype and phenotype data, macromolecular structure, gene expression data, or crystallographic data were generated.
