# Supplementary material for: Extracranial arteriovenous malformations demonstrate dysregulated TGF-β/BMP signaling and increased circulating TGF-β1
Source: Sci Rep. 2022 Oct 5;12:16612. doi: 10.1038/s41598-022-21217-0 (PMC9534897; doi:10.1038/s41598-022-21217-0)
Supplement: Supplementary file 1 — Supplementary Figures. [file 41598_2022_21217_MOESM1_ESM.pptx]

## Slide 1
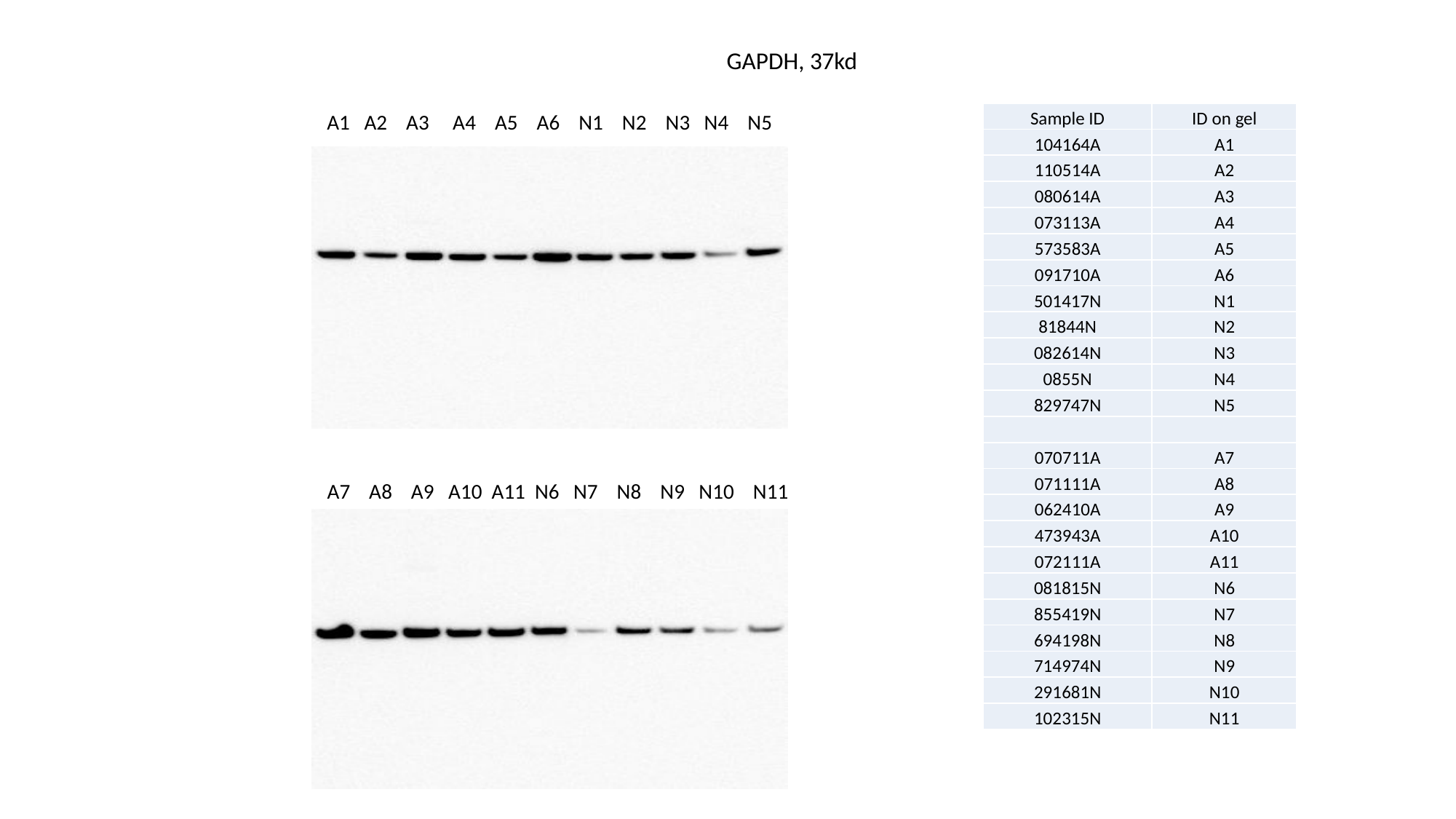

GAPDH, 37kd
 A1 A2 A3 A4 A5 A6 N1 N2 N3 N4 N5
| Sample ID | ID on gel |
| --- | --- |
| 104164A | A1 |
| 110514A | A2 |
| 080614A | A3 |
| 073113A | A4 |
| 573583A | A5 |
| 091710A | A6 |
| 501417N | N1 |
| 81844N | N2 |
| 082614N | N3 |
| 0855N | N4 |
| 829747N | N5 |
| | |
| 070711A | A7 |
| 071111A | A8 |
| 062410A | A9 |
| 473943A | A10 |
| 072111A | A11 |
| 081815N | N6 |
| 855419N | N7 |
| 694198N | N8 |
| 714974N | N9 |
| 291681N | N10 |
| 102315N | N11 |
A7 A8 A9 A10 A11 N6 N7 N8 N9 N10 N11

## Slide 2
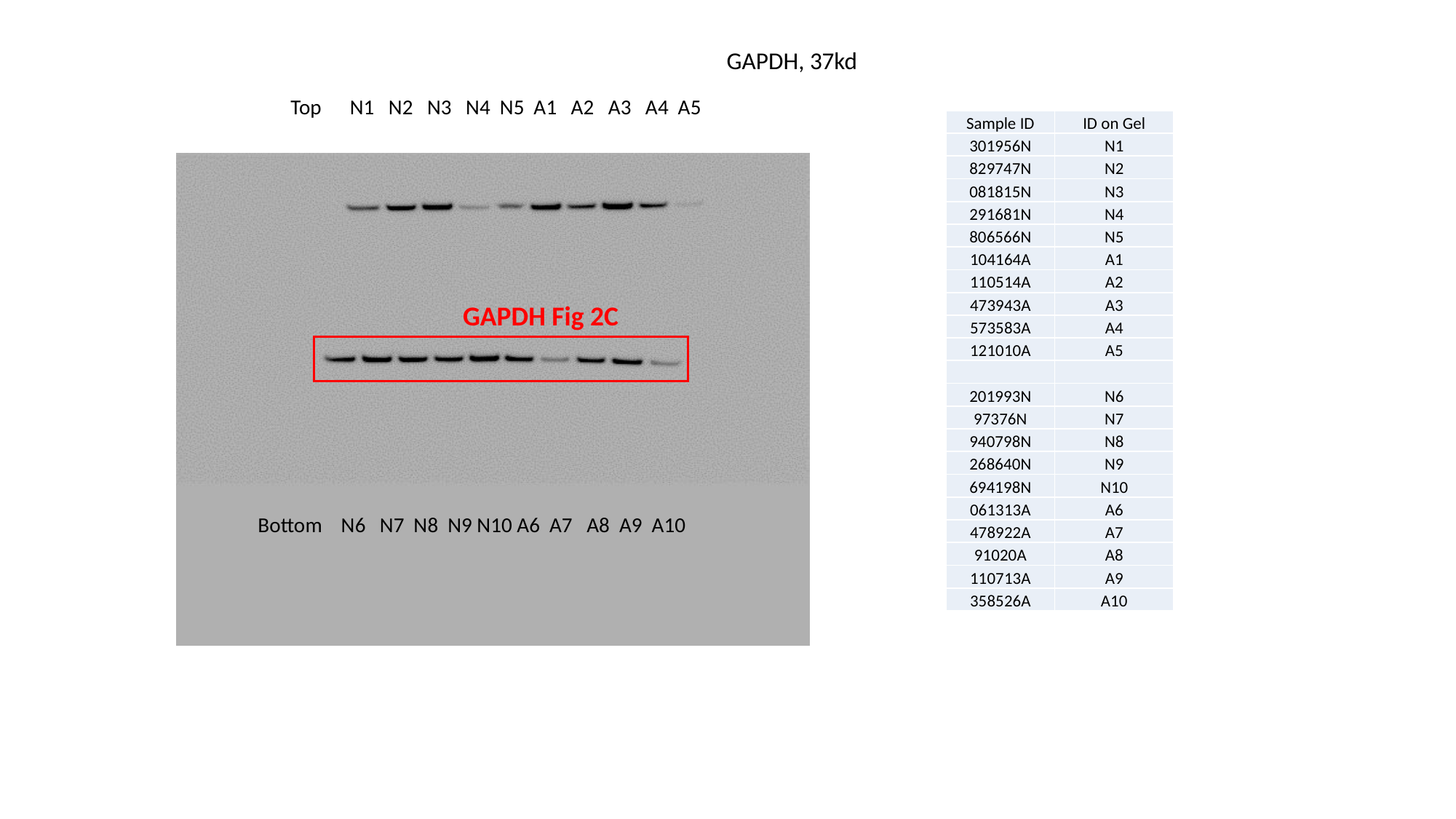

GAPDH, 37kd
Top N1 N2 N3 N4 N5 A1 A2 A3 A4 A5
| Sample ID | ID on Gel |
| --- | --- |
| 301956N | N1 |
| 829747N | N2 |
| 081815N | N3 |
| 291681N | N4 |
| 806566N | N5 |
| 104164A | A1 |
| 110514A | A2 |
| 473943A | A3 |
| 573583A | A4 |
| 121010A | A5 |
| | |
| 201993N | N6 |
| 97376N | N7 |
| 940798N | N8 |
| 268640N | N9 |
| 694198N | N10 |
| 061313A | A6 |
| 478922A | A7 |
| 91020A | A8 |
| 110713A | A9 |
| 358526A | A10 |
GAPDH Fig 2C
Bottom N6 N7 N8 N9 N10 A6 A7 A8 A9 A10

## Slide 3
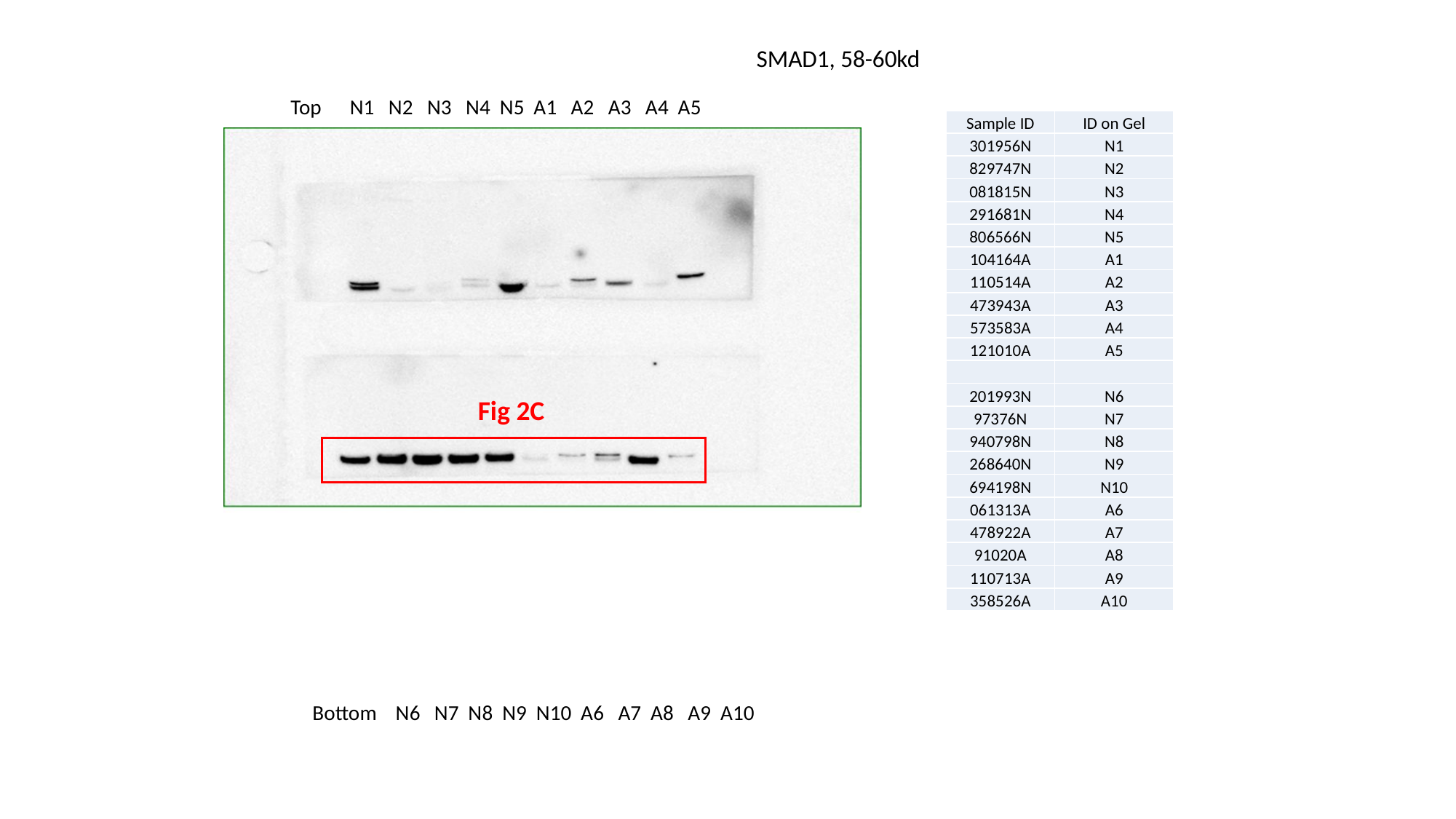

SMAD1, 58-60kd
Top N1 N2 N3 N4 N5 A1 A2 A3 A4 A5
| Sample ID | ID on Gel |
| --- | --- |
| 301956N | N1 |
| 829747N | N2 |
| 081815N | N3 |
| 291681N | N4 |
| 806566N | N5 |
| 104164A | A1 |
| 110514A | A2 |
| 473943A | A3 |
| 573583A | A4 |
| 121010A | A5 |
| | |
| 201993N | N6 |
| 97376N | N7 |
| 940798N | N8 |
| 268640N | N9 |
| 694198N | N10 |
| 061313A | A6 |
| 478922A | A7 |
| 91020A | A8 |
| 110713A | A9 |
| 358526A | A10 |
Fig 2C
Bottom N6 N7 N8 N9 N10 A6 A7 A8 A9 A10

## Slide 4
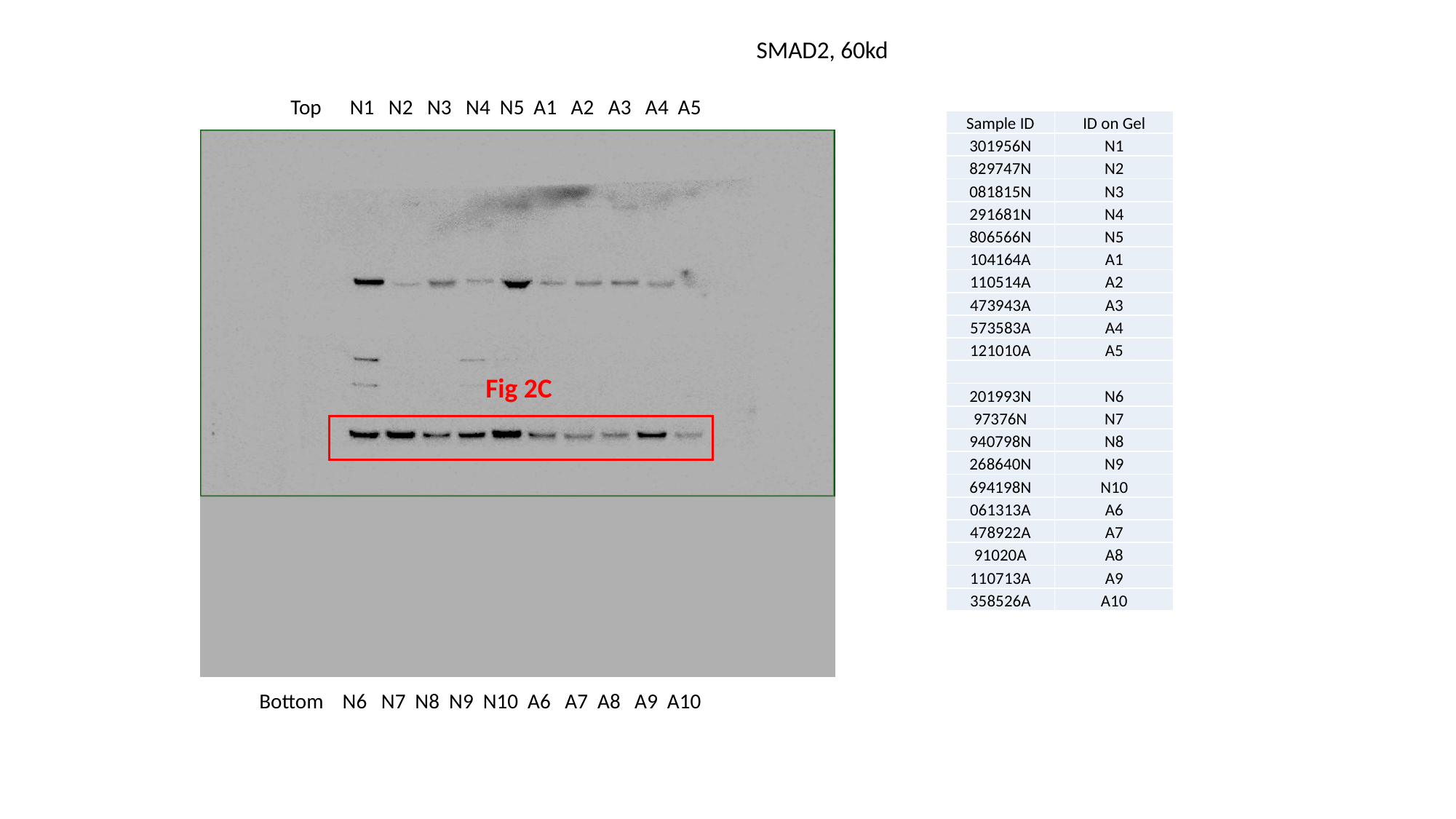

SMAD2, 60kd
Top N1 N2 N3 N4 N5 A1 A2 A3 A4 A5
| Sample ID | ID on Gel |
| --- | --- |
| 301956N | N1 |
| 829747N | N2 |
| 081815N | N3 |
| 291681N | N4 |
| 806566N | N5 |
| 104164A | A1 |
| 110514A | A2 |
| 473943A | A3 |
| 573583A | A4 |
| 121010A | A5 |
| | |
| 201993N | N6 |
| 97376N | N7 |
| 940798N | N8 |
| 268640N | N9 |
| 694198N | N10 |
| 061313A | A6 |
| 478922A | A7 |
| 91020A | A8 |
| 110713A | A9 |
| 358526A | A10 |
Fig 2C
Bottom N6 N7 N8 N9 N10 A6 A7 A8 A9 A10

## Slide 5
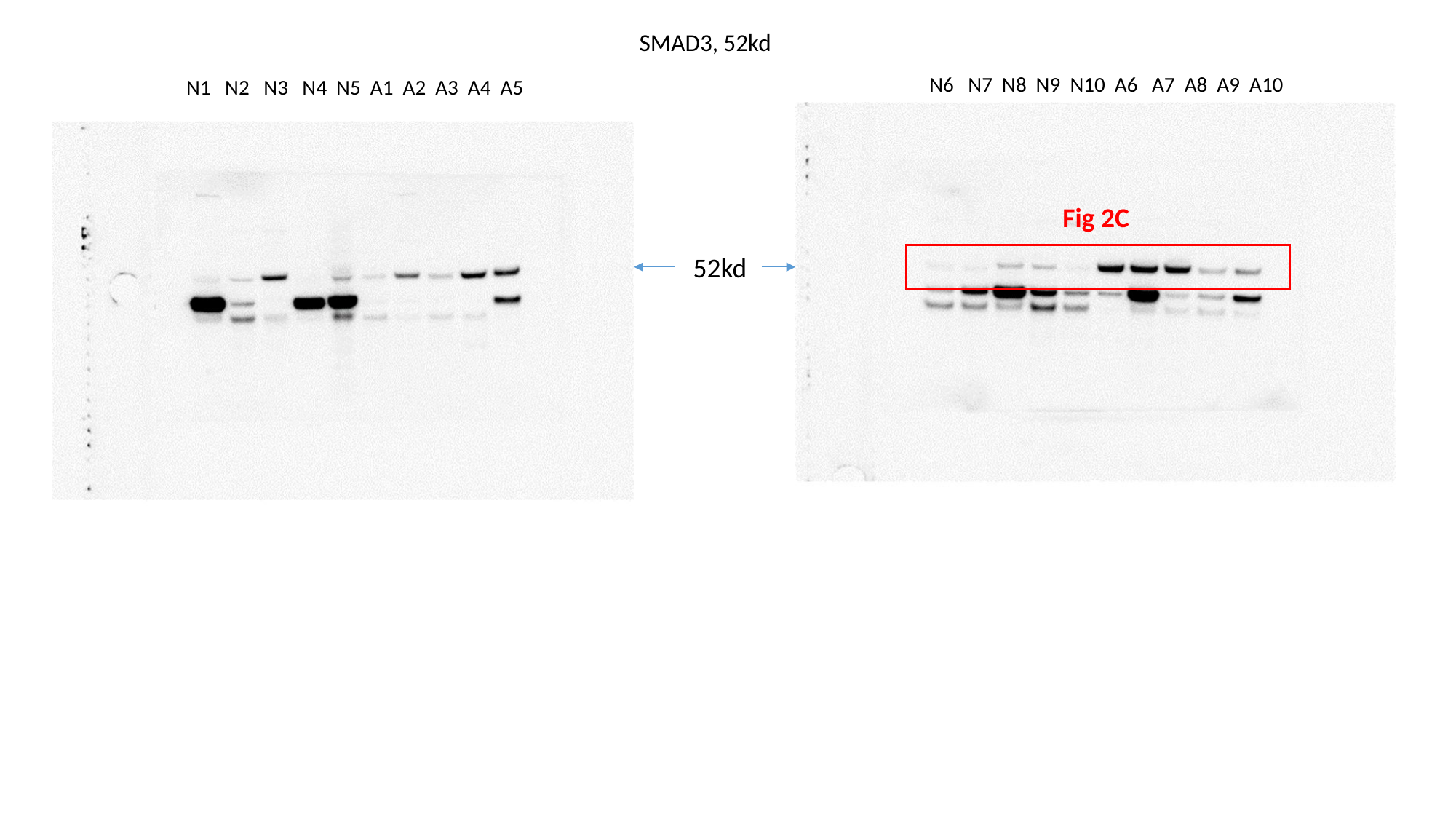

SMAD3, 52kd
N6 N7 N8 N9 N10 A6 A7 A8 A9 A10
N1 N2 N3 N4 N5 A1 A2 A3 A4 A5
Fig 2C
52kd

## Slide 6
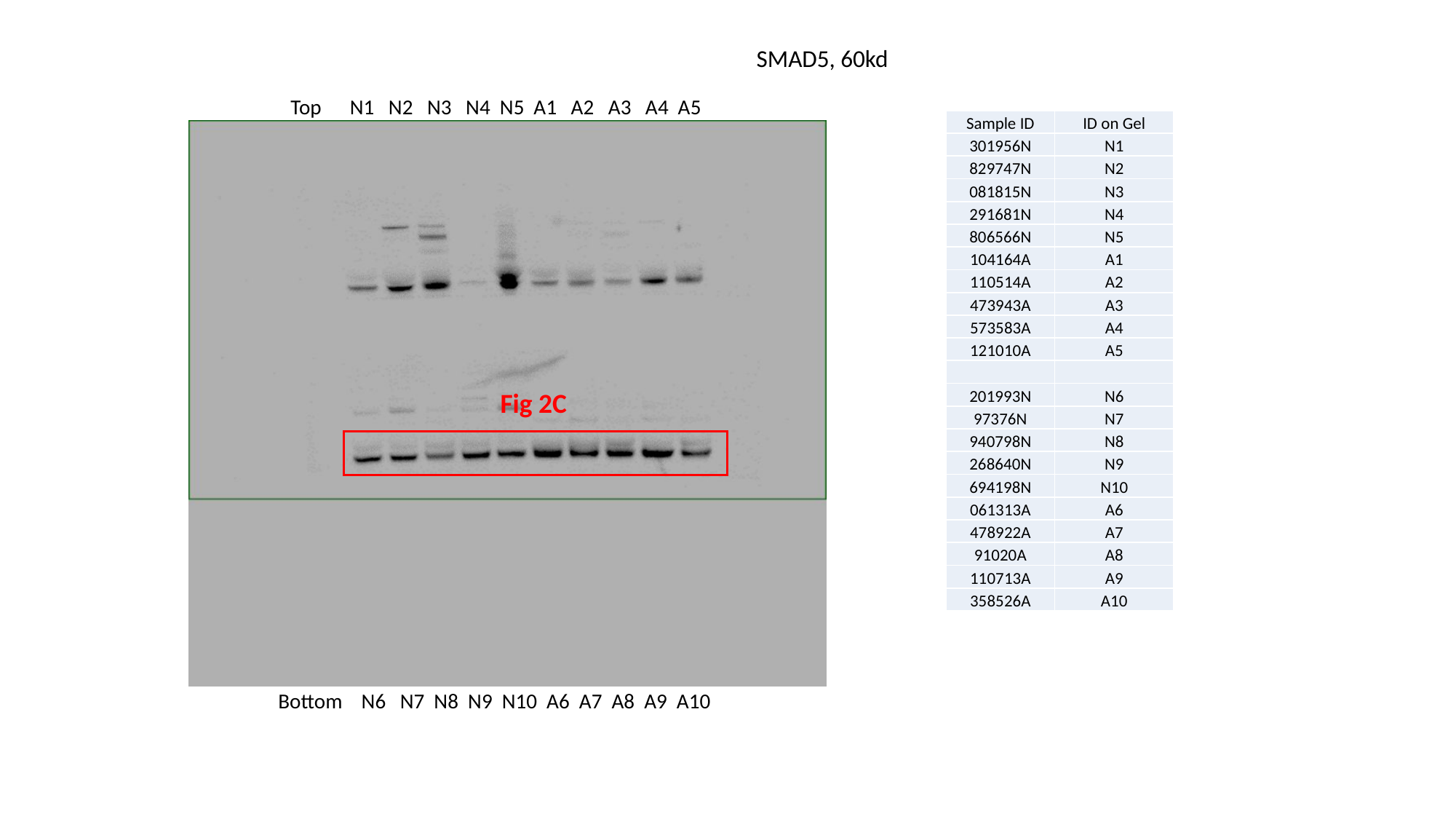

SMAD5, 60kd
Top N1 N2 N3 N4 N5 A1 A2 A3 A4 A5
| Sample ID | ID on Gel |
| --- | --- |
| 301956N | N1 |
| 829747N | N2 |
| 081815N | N3 |
| 291681N | N4 |
| 806566N | N5 |
| 104164A | A1 |
| 110514A | A2 |
| 473943A | A3 |
| 573583A | A4 |
| 121010A | A5 |
| | |
| 201993N | N6 |
| 97376N | N7 |
| 940798N | N8 |
| 268640N | N9 |
| 694198N | N10 |
| 061313A | A6 |
| 478922A | A7 |
| 91020A | A8 |
| 110713A | A9 |
| 358526A | A10 |
Fig 2C
Bottom N6 N7 N8 N9 N10 A6 A7 A8 A9 A10

## Slide 7
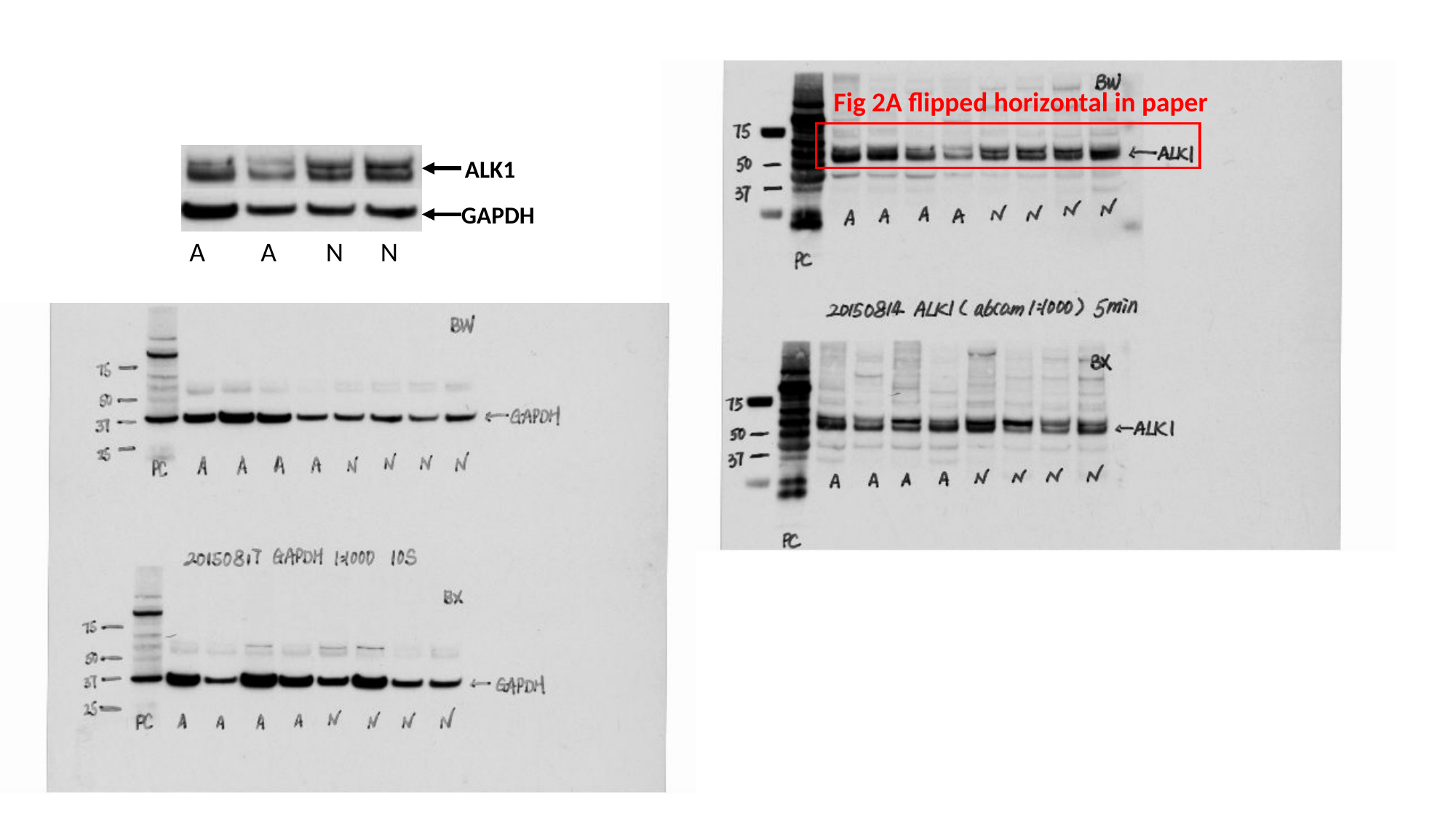

Fig 2A flipped horizontal in paper
ALK1
GAPDH
 A A N N

## Slide 8
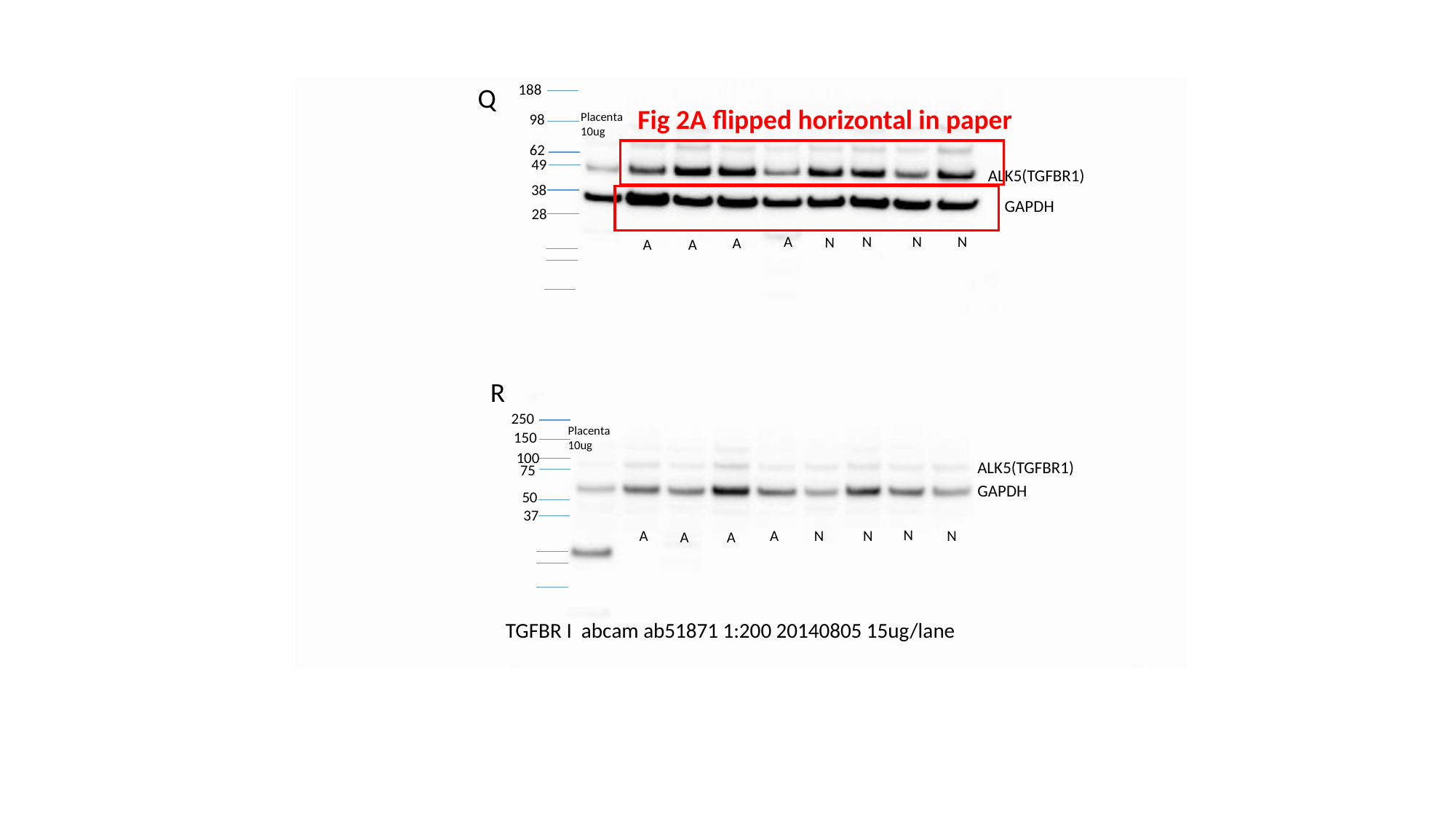

188
Q
Fig 2A flipped horizontal in paper
98
Placenta 10ug
62
49
ALK5(TGFBR1)
38
GAPDH
28
N
N
A
N
N
A
A
A
R
250
Placenta 10ug
150
100
ALK5(TGFBR1)
75
GAPDH
50
37
N
N
N
A
N
A
A
A
TGFBR I abcam ab51871 1:200 20140805 15ug/lane

## Slide 9
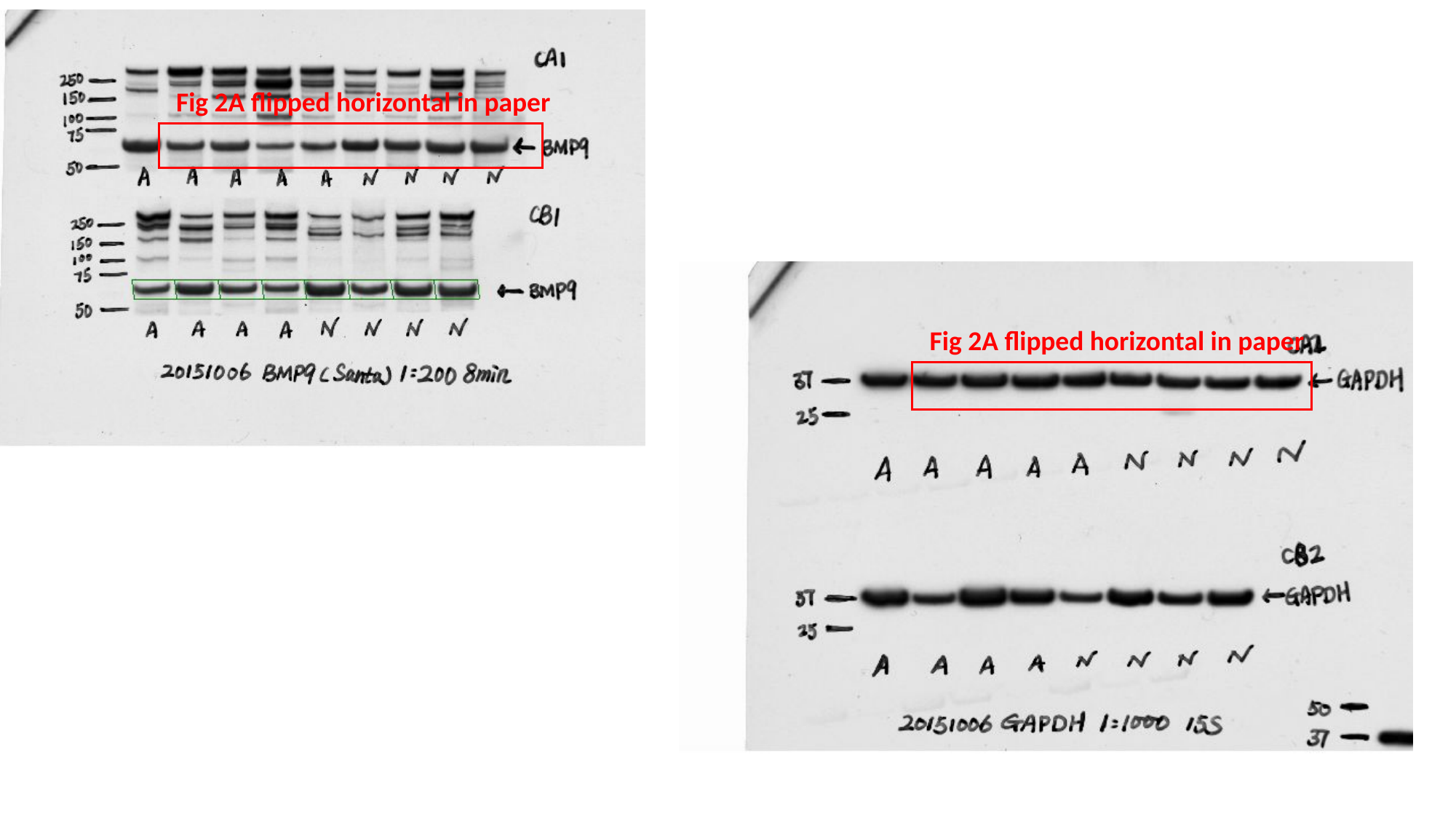

Fig 2A flipped horizontal in paper
Fig 2A flipped horizontal in paper

## Slide 10
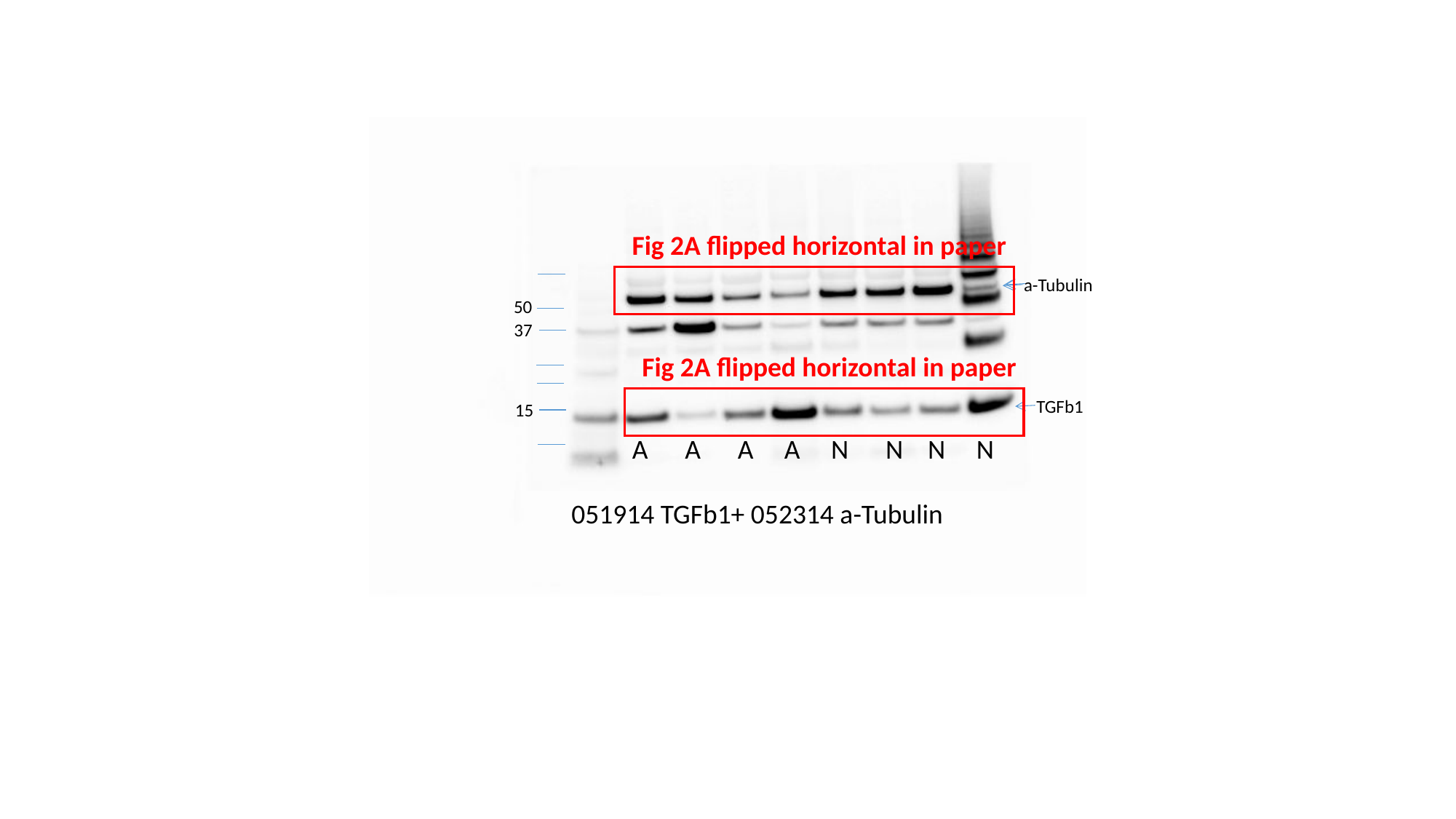

Fig 2A flipped horizontal in paper
a-Tubulin
50
37
Fig 2A flipped horizontal in paper
TGFb1
15
A A A A N N N N
051914 TGFb1+ 052314 a-Tubulin

## Slide 11
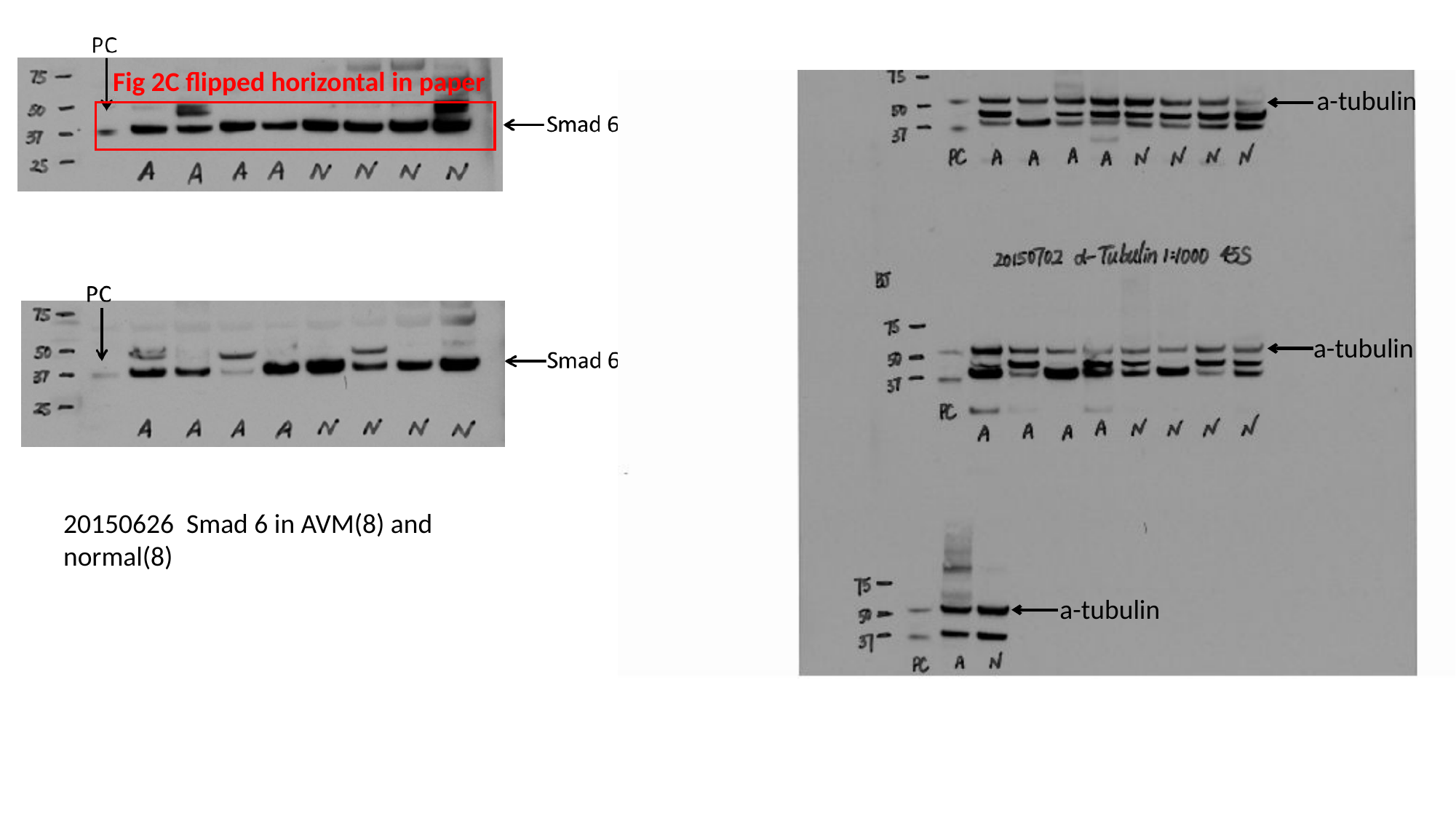

20150626 Smad 6 in AVM(8) and normal(8)
Fig 2C flipped horizontal in paper
a-tubulin
a-tubulin
a-tubulin

## Slide 12
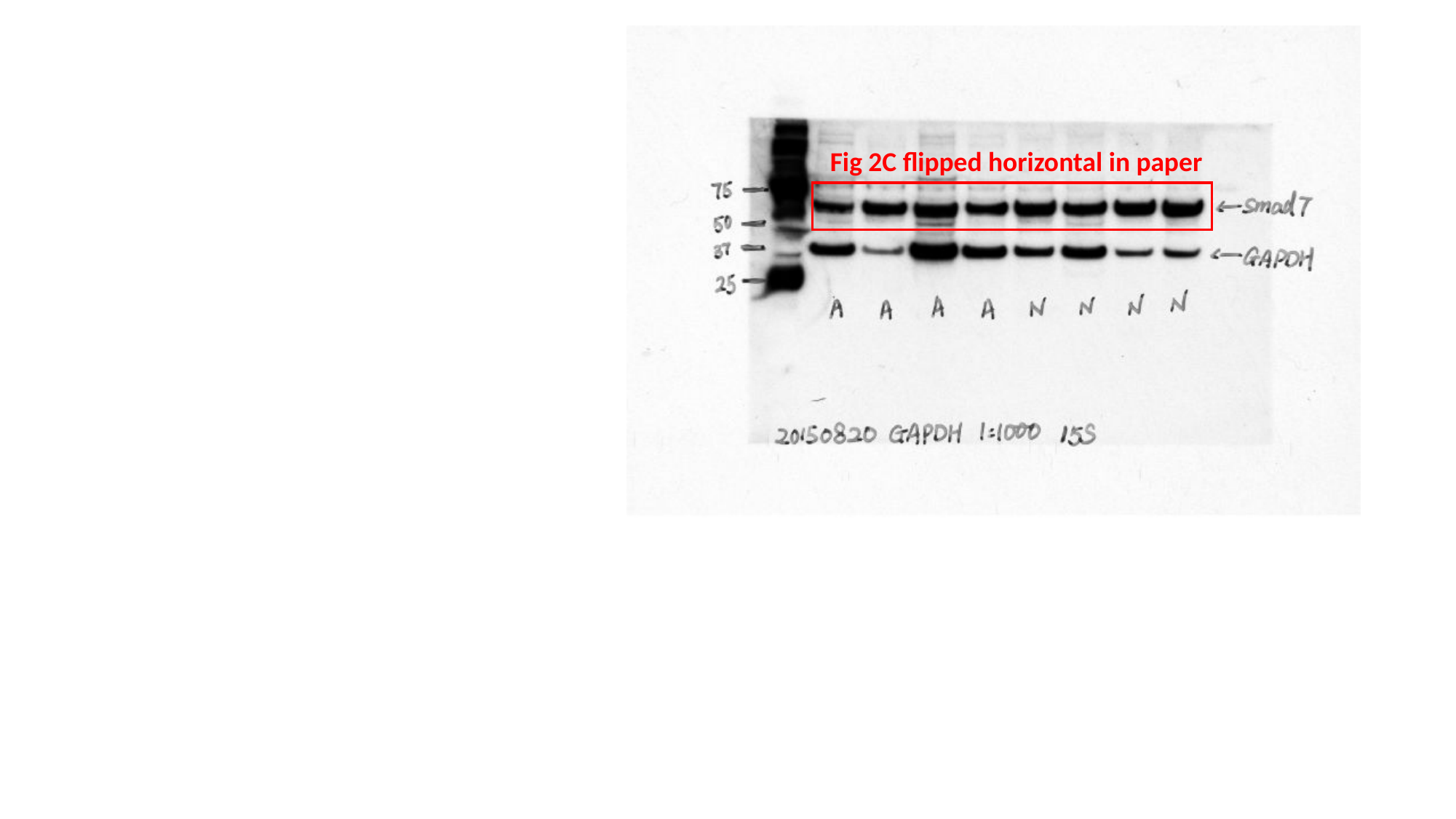

Fig 2C flipped horizontal in paper

## Slide 13
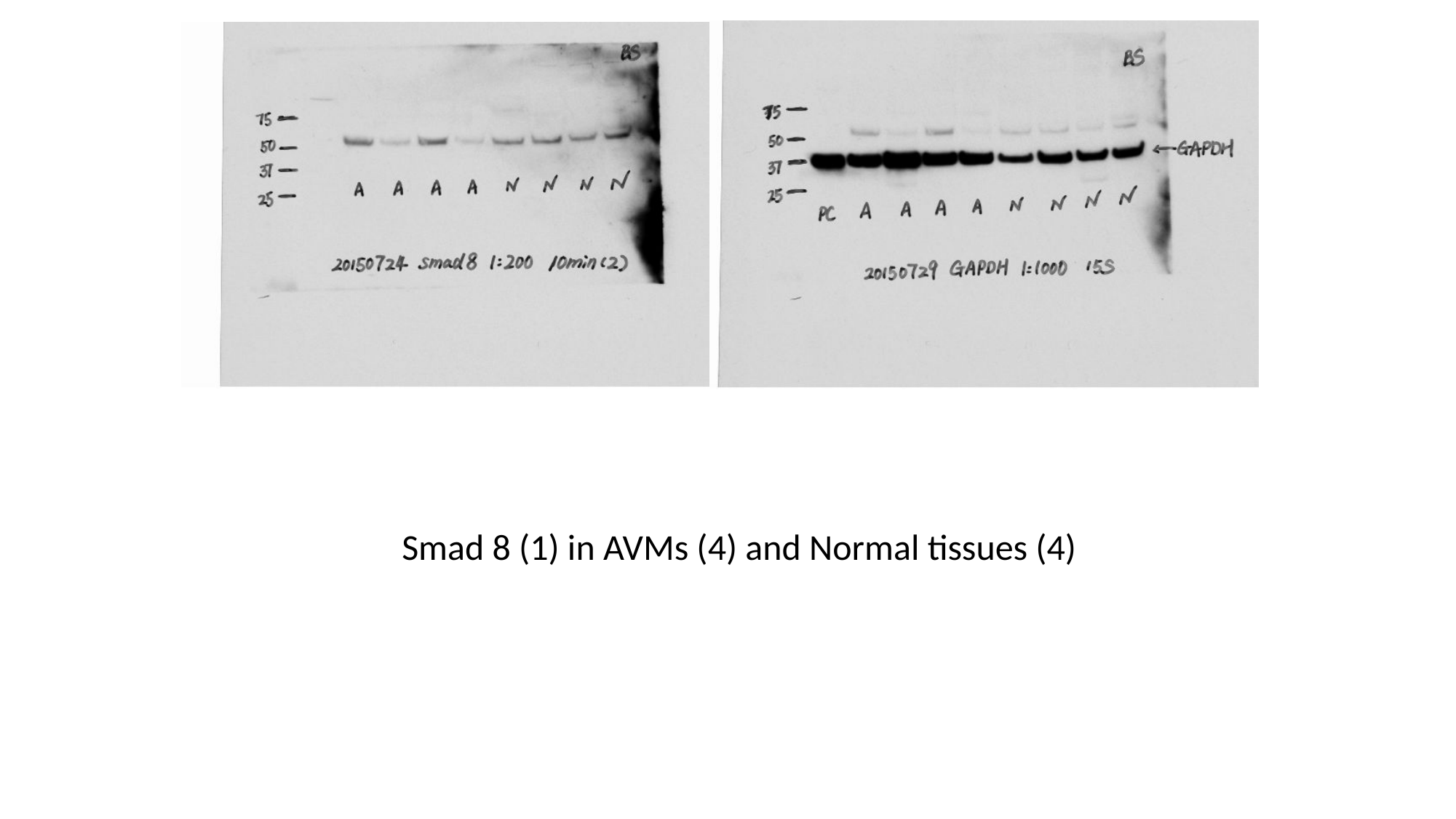

Smad 8 (1) in AVMs (4) and Normal tissues (4)

## Slide 14
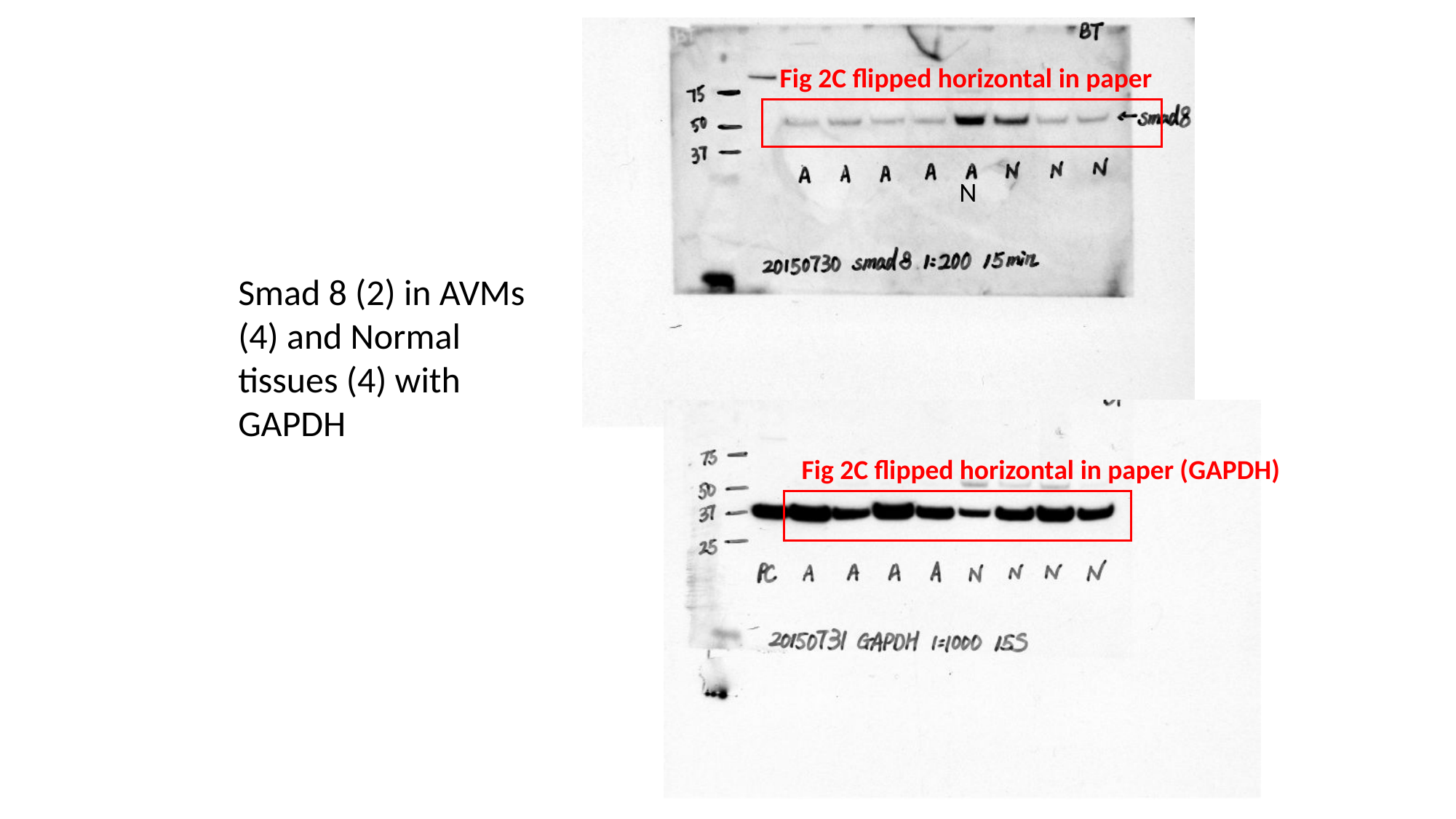

Fig 2C flipped horizontal in paper
N
Smad 8 (2) in AVMs (4) and Normal tissues (4) with GAPDH
Fig 2C flipped horizontal in paper (GAPDH)
